# Gender Differences in Food Choice: Effects of Superior Temporal Sulcus Stimulation

**DOI:** 10.3389/fnhum.2017.00597

**Published:** 2017-12-07

**Authors:** Valerio Manippa, Caterina Padulo, Laura N. van der Laan, Alfredo Brancucci

**Affiliations:** ^1^Department of Neuroscience, Imaging and Clinical Sciences, University “G. d’Annunzio” of Chieti-Pescara, Chieti, Italy; ^2^Department of Psychological Sciences, Health, and the Territory, University “G. d’Annunzio” of Chieti-Pescara, Chieti, Italy; ^3^Image Sciences Institute, University Medical Center Utrecht, Utrecht, Netherlands

**Keywords:** food evaluation, food choice, calorie, sex differences, transcranial random noise stimulation, superior temporal sulcus

## Abstract

The easy availability of food has caused a shift from eating for survival to hedonic eating. Women, compared to men, have shown to respond differently to food cues in the environment on a behavioral and a neural level, in particular to energy rich (compared to low energy) foods. It has been demonstrated that the right posterior superior temporal sulcus (STS) is the only region exhibiting greater activation for high vs. low calorie food choices. In order to test for a possible causal role of STS in food choice, we applied high frequency transcranial random noise stimulation (tRNS) on STS assuming a different response pattern between males and females. Our participants (18 females, 17 males) performed a forced choice task between food pairs matched for individual liking but differed in calorie, during the left STS, right STS stimulation and sham condition. Male participants showed a general preference for low calorie (LC) foods compared to females. In addition, we observed in males, but not in females, an increase of high calorie (HC) food choice during right STS tRNS compared to sham condition and left STS tRNS. Finally, we found an increase of missed choices during right STS stimulation compared to sham condition and left STS stimulation. In conclusion, thanks to tRNS evidence, we both confirm the involvement and suggest a causal role of right posterior STS in feeding behavior. Moreover, we suggest that gender differences exist in STS mechanisms underlying food choice.

## Introduction

The easy availability of food in our western society has caused a shift from eating for survival (energy intake) to an hedonic eating aimed to obtain pleasant feelings (reward) from food intake (Peters et al., 2002; Saper et al., 2002; Mela, 2006). The consequent acquisition of incorrect food habits (Epstein and Leddy, 2006), i.e., favoring high energy foods, could exert negative effects on human well-being (Shepherd and Dennison, 1996), increasing the risk to weight gain and, consequently, overweight and obesity (McLellan, 2002). Therefore, in the last decades many studies have focused on feeding behavior and food decision making (e.g., Drewnowski, 1997; Wansink, 2004; Friese et al., 2008; Vabø and Hansen, 2014). It seems that, in addition to the psycho-physiological condition (e.g., hunger level: de Castro, 1988; Finlayson et al., 2007), the main factors that modulate food intake are the sex of the eaters (e.g., Wardle et al., 2004; Arganini et al., 2012) and the meal-related pleasure experienced (e.g., Eertmans et al., 2001; Drewnowski and Almiron-Roig, 2009).

Generally, the most palatable and, therefore, pleasurable foods are both energy dense and high in fat content. Effectively, compared to low-calorie (LC) food, such as fruit and vegetables, the preference for high-calorie (HC) foods (Drewnowski, 1997; Wansink et al., 2003; Drewnowski et al., 2012) depends on its higher sensory pleasure and positive metabolic/physiological post-ingestive consequences such as satiety and a pleasurable state (Birch, 1999; Wansink et al., 2003). Effectively, the simple exposure to food pictures elicits anticipatory responses similar to food intake (Berridge, 1999; Dagher, 2012; Huerta et al., 2014) and HC compared to LC food viewing results in increased activation of the meso-limbic-cortical reward circuit (Frank et al., 2010; van der Laan et al., 2011).

Women, compared to men, have been shown to respond differently to foods. In fact, it has been reported that women tend to be more invested in food-related issues, have better knowledge of food and nutrition, are more prone to go on a diet, and are more likely to perceive themselves as needing to lose weight (Pingitore et al., 1997; Neumark-Sztainer et al., 1999; Davy et al., 2006). For example, Uccula and Nuvoli (2017) showed that women, more than men, tend to overestimate their weight and thus to decrease their meal. More generally, women are reported to have higher intakes of fruit and vegetables, higher intakes of dietary fiber and lower intakes of fat and salt, conferring greater importance to healthy eating (Wardle et al., 2004; Arganini et al., 2012). These tendencies seem to show a corresponding neural pattern: there are well-documented differences between the sexes in the organization and structure of the brain particularly regarding areas related to neurocognitive functioning (Cahill, 2006; Luders et al., 2009) and food-reward processing (Del Parigi et al., 2002; Smeets et al., 2006; Haase et al., 2011). Killgore and Yurgelun-Todd (2010) found that the viewing of HC food elicited a different brain activation between men and women. Particularly, compared to men, women showed significantly greater activation within dorsolateral, ventrolateral and ventromedial prefrontal cortex, middle/posterior cingulate and insula, which are cortical regions involved in behavioral control and self-referential cognition.

Notwithstanding the growing number of neuroimaging studies aiming to investigate the neural correlates of food choices (e.g., Plassmann et al., 2010; Levy and Glimcher, 2011; van der Laan et al., 2012), till date no studies have investigated brain responses during forced choices between high and low energy foods. Recently Charbonnier et al. (2015), examined brain responses during food choices between equally valenced high and low energy foods, and non-food choices in sated participants. Results revealed stronger activation in the left insula, superior temporal sulcus (STS), posterior cingulate gyrus and precuneus for food choice compared to non-food choice, suggesting that the food stimuli were more salient despite subject’s low motivation to eat. Particularly, the right posterior STS was the only region that exhibited greater activation when participants chose HC food compared to LC ones. This suggests that the right posterior STS activation may reflect food’s biological relevance independently from food preference (Piech et al., 2010). This area is involved in different psychological processes (e.g., attention, face processing, social cognition) other than in food perception, it is linked to prefrontal and mesolimbic regions (Hein and Knight, 2008) which have both a crucial role in sex-dependent differences in food processing (Chao et al., 2017).

However, no study has confirmed the role of STS in feeding behavior considering the differences between men and women. Non-invasive neuromodulation approaches as transcranial magnetic stimulation and transcranial direct-current stimulation are used to study basic mechanisms underlying eating behavior and to treat its disorders. However, most of these studies mainly involved the stimulation of dorsolateral prefrontal cortex in female subjects, with the purpose of assessing possible effects on food craving (e.g., Uher et al., 2005; Fregni et al., 2008) or food evaluation (Camus et al., 2009). In this field a novel method of electrical stimulation is the transcranial random-noise stimulation (tRNS) in which a random amplitude electrical current is applied over the scalp for several seconds to minutes (Terney et al., 2008; Paulus, 2011). Particularly high-frequency (HF) tRNS seems to be responsible for generating an excitability increase (Francis et al., 2003; Terney et al., 2008) and a reduction of blood-oxygen-level-dependent response in motor tasks (Chaieb et al., 2009; Saiote et al., 2013). Fertonani et al. (2011) suggested that the mechanism of action of HF-tRNS can prevent the homeostasis of the system and reinforce the task-related neural activity, showing its superiority in comparison to other transcranial electrical stimulation techniques (e.g., Prete et al., 2017). Despite that, tRNS has been mainly used during motor and perceptive-learning tasks involving the (pre)frontal cortex stimulation. No-study, indeed, has been conducted with the purpose to study the possible effects of the STS stimulation during the execution of a food choice task.

Thus, our aim was to investigate how males and females differ in neural mechanisms underlying food choice. To this end, we applied tRNS on STS to evaluate potential neuromodulation effects on forced choices of equally valenced high and LC food pairs in sated participants which would suggest a causal role of the stimulated site and not a collateral activity. In line with Charbonnier et al. (2015) findings, we expect that the right STS stimulation results in an increase of stimuli salience during choice and, thus, in augmented HC food choice. Moreover, according to the reported gender differences in the neural processing of HC food, we expect a different response pattern between males and females.

## Materials and Methods

### Participants

By means of a telephone interview, we initially recruited 47 young adult participants with a mean age of 21.3 ± 3.0 (standard deviation, SD) years old, 25 females and 22 males normal-weight with a mean BMI of 22.8 (SD = ±2.1). All participants were right-handed. Only non-smokers subjects were recruited reporting no drug abuse history, no diagnosis of psychiatric and neurological illness and no metallic implants/implanted electric devices. All participants completed a screening package aimed to check the absence of eating disorders. Other, no one followed special diets or had dietary restrictions, due to allergies, intolerances, or vegetarianism.

In order to bring all participants to a comparable sated state (measured by means of a paper-and-pencil test; see Foroni et al., 2013), they were requested to eat until satiety the same meal before both tasks. Moreover, they were asked to avoid the use of drugs medicine and alcohol in the 24 h prior to the experiment. Food pairs presented in the food choice task were based on participants’ ratings given in the food picture rating task. Participants for whom less than 45 choice pairs could be constructed, were excluded from the tRNS task (*N* = 11). Another participant did not want to attend the second task. The final sample of 17 males and 18 females did not show significant differences in age and BMI.

### Stimuli

We used 183 colored food images taken from Full4Health Image Collection (University Medical Center Utrecht; Charbonnier et al., 2016)^1^. These foods can be divided into two categories: 92 HC (mean Kcal/100 g = 457, SD = ±77) and 91 LC (mean Kcal/100 g = 76; SD = 53) images including both sweet and savory items. Each food was presented on a plate. The plates were full and were presented on a standardized background.

### Procedure

The whole procedure was carried out in accordance with the principles of the Declaration of Helsinki; the protocol was approved by the Research Ethics Committee, University of Chieti-Pescara, and participants gave written and informed consent before beginning the experiments.

The rating task and the forced choice task were implemented by e-Prime 1.1 (Psychology Software Tools) and administered in two different days. In particular, participants performed the forced choice task at least 7 days after the rating task. Participants were tested individually: they sat comfortably in front of the computer monitor (19 inch, 1600 × 1200 pixel) with the head at approximately 60 cm distance. First, participants’ height and weight were measured in order to assess the BMI, and they were asked to report their sex and age. Then, the experimenter instructed the participants and left the experimental room.

Before the rating task, participants completed a screening package that included the Italian version of the Eating Attitude Test (EAT-26; Garner et al., 1982), the Binge Eating Scale (BES; Gormally et al., 1982), the Body Uneasiness Test part A (BUT-A; Cuzzolaro et al., 2006) and the Dutch Eating Behavioral Questionnaire (DEBQ; van Strien et al., 1986). This was made in order to exclude possible eating disorders and to assess individuals’ eating patterns and body issues. Finally, we assessed participants’ laterality preference using the Edinburgh Handedness Inventory (EHI; Oldfield, 1971) to ensure about their right-handedness. Male and female samples showed significant differences (independent groups *t*-test) in the BUT-A’s General Severity Index and in the Emotional Eating subscale of DEBQ scores, both higher in females compared to the males (these are well-known gender-related differences, see e.g., van Strien et al., 1986; Pingitore et al., 1997; Marano et al., 2007; Dakanalis et al., 2013). They were comparable for all the other scores (see Table 1).

**Table 1:** Mean and standard deviation of questionnaire scores.

	Female	Male
	Mean ± SD	Mean ± SD
Eating attitude test-26	3.72 ± 2.67	2.88 ± 3.08
Body uneasinnes test-A	*1.04 ± 0.77	*0.45 ± 0.44
Binge eating scale	5.39 ± 3.66	4.76 ± 4.93
DEBQ-restrained eating	2.18 ± 0.73	1.96 ± 0.81
DEBQ-emotional eating	*2.06 ± 0.73	*1.31 ± 0.39
DEBQ-external eating	3.00 ± 0.52	2.61 ± 0.61
Edinburgh handedness inventory	87.65 ± 21.21	93.78 ± 14.67

*p < 0.05 (Female vs. Male).

Half of the participants, for each gender, performed both tasks in the morning (after breakfast) and the other half in the afternoon (after lunch). Each participant was asked to eat the same meal until satiety before both experiments. Specifically, they reported the meal consumed before starting the rating task and then, prior to the forced choice task, we requested them to eat the same foodstuffs. We evaluated satiety level, before both tasks, using the same paper-and-pencil test performed in the demographic phase of FRIDa’s validation (Foroni et al., 2013). This test was composed by five queries assessing the psycho-physiological state of the participants (i.e., hunger, thirstiness, tiredness). Responses were given on a 0–100-point scale indicating a point on a line with differently-labeled extremities. Particularly, we excluded participants who reported scores greater than 30 (Padulo et al., 2017) to the following questions: “How hungry are you now?” (from 0 “not at all hungry” to 100 “very hungry”), “How much time did pass since your last complete meal?” (from 0 “less than an hour” to 100 “more than 5 h”). Our male and female samples showed no differences in the satiety levels.

#### Food Picture Rating Task

**Figure 1:**
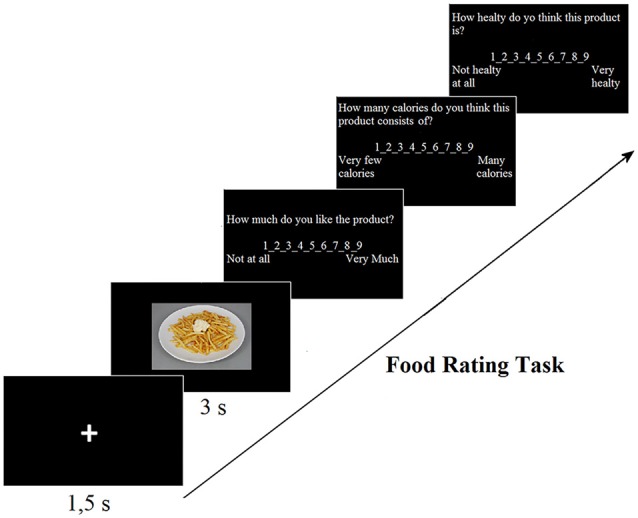
Trial sequence in the food rating task.

The food picture rating task was based on the Leeds Food Preference Questionnaire (LFPQ; Finlayson et al., 2007). During this task participants rated liking, caloric content and healthiness of 183 food pictures (726 × 486 pixel) on a 9-point Likert scale (see Figure 1). Participants received the following instructions (in Italian): “You will see 183 product pictures. You have to respond to three questions about the liking, the calorie and the healthiness of each product. Try to answer as quickly as possible. There are no correct or incorrect answers, it’s about your opinion. Don’t think too long about an answer, the first you think is usually the best one”. Then they were left alone in the experimental room. Each trial started with a fixation cross of 1.5 s, followed by the food picture showed for 3 s. Just after each picture participants were asked to answer the following questions: “How much do you like the product?’ (1 not at all—9 very much), “How many calories do you think this product consists of?” (1 very few calories—9 many calories) and “How healthy do you think this product is?” (1 not healthy at all—9 very healthy). The rating task lasted about 30 min and was divided into two blocks, separated by a pause. Both the order of presentation of the food pictures and the three questions were randomized between participants.

#### Forced Choice tRNS Task

Based on the ratings collected during the food picture rating task, food pairs were created for each participant. Food pairs (544 × 364 pixel) were matched on liking (i.e., equal ratings) and taste (i.e., sweet or savory), to make the pairs as equal as possible. They differed in perceived healthiness, absolute and perceived caloric content. To check whether our manipulations were successful, mean actual caloric content (in kcal × 100 g), mean liking, perceived caloric content, and healthiness (using a 9-point Likert scale) were calculated. As expected, all variables, except for liking, differed significantly between the choice options within each food pair (Table 2). Hence, the study manipulations were effective. Participants with a minimum of 45 food pairs were enrolled in tRNS task. Food pairs (ranged from 48 to 77) were 61.9 ± 8.0 for males and 62.3 ± 9.4 for females (mean ± SD).

**Table 2:** Mean and standard deviation of food rating task.

	High calorie foods	Low calorie foods
	Mean ± SD	Mean ± SD
Liking	6.06 ± 1.20	6.03 ± 1.11
Perceived calorie	*6.57 ± 1.08	*4.07 ± 1.16
Healthy	*3.74 ± 1.06	*7.00 ± 0.80
Real calorie	*457 ± 77	*76 ± 53

All the variables ranged from 1 to 9 on a Likert scale excepted for Real Calorie (Kcal × 100 g of product). *p < 0.05 (High Calorie vs. Low Calorie).

**Figure 2:**
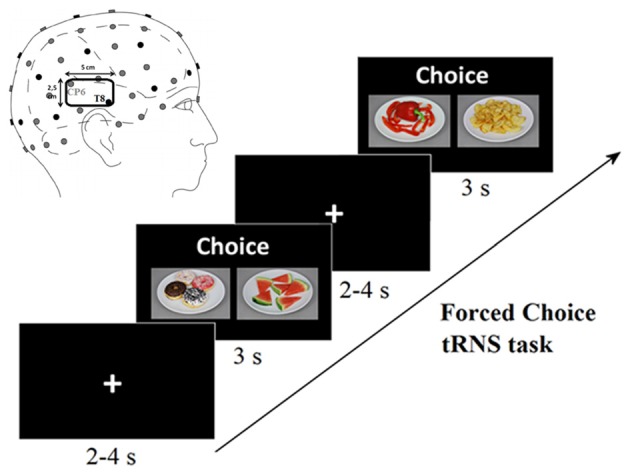
Two consecutive examples of forced choice task trials carried out during high-frequency (HF) transcranial random noise stimulation (tRNS) conditions. In the 3 s lapse of food pairs presentation, participants had to make their choice by key press. Top left: position of the right superior temporal sulcus (STS) electrode, the reference electrode was placed on the left shoulder (and vice versa for left STS electrode).

Forced choice tRNS task (Figure 2) consisted in three blocks of choice between two foods. Each block corresponded to one of the three conditions: left posterior STS (lSTS) stimulation, sham (SH) as control condition and right posterior STS (rSTS) stimulation counterbalanced across participants. At the beginning of the session, the experimenter gave the following instruction in Italian: “Choose the product of which you most want to eat at this moment”. In addition to the verbal instruction, the label “Choice” was shown above every choice pair. Participants had 3 s to indicate their choice by a key press (“G” key for the food on the left side, “L” key for the food on the right side), whenever they failed to make a choice within the restricted time, the event was labeled as a missed choice. After each choice a fixation cross of variable duration (2–4 s), was presented. In each block the food pairs were the same, but were presented in a pseudorandom order. This task had a duration of approximately 30 min.

tRNS (Terney et al., 2008) was delivered by a battery-driven (Eldith DC—Stimulator Plus, NeuroConn GmbH, Germany) through a pair of surface saline-soaked sponge electrodes kept firm by elastic bands. A HF random noise current (100–640 Hz) of 1.5 mA was applied through an electrode 2.5 × 5 cm (resulting in a current density of 0.120 mA/cm^2^) placed at the T7-CP5 or T8-CP6 positions of the 10–10 EEG system (Koessler et al., 2009) corresponding to left/right posterior STS (see Figure 2). The reference electrode, 5 × 7 cm, was placed on the contralateral shoulder (resulting in a current density of 0.043 mA/cm^2^). The stimulation started 150 s before the beginning of the task. During the task the stimulation lasted from a variable time between a minimum of 278 s to a maximum of 488 s (mean on-line stimulation time = 363.4 s, SD = ±30.4 s) depending on the trials number (ranged from 48 to 77) and the response times of our participants. A ramping period of 15 s both at the beginning and at the end of the stimulation was also applied. Thus, the total stimulation time ranged from ~450 s to ~650 s (i.e., 15 s fade-in, 150 s before the beginning of the task, a period varying from 278 s and 488 s during the task and 15 s fade-out). In the SH control condition, the electrode was placed on one of the two stimulation sites (balanced between participants), and the current was turned off after 15 s. The stimulation conditions (left, right STS and SH) order were counterbalanced across participants. There was a break of 360 s between blocks.

### Data Analysis

Data analysis was conducted using Statistica 8.0 (StatSoft). Dependent variables in the tRNS forced choice task were the percentage of HC food choices, the median of reaction times (RTs) for both HC and LC, and the percentage of missed choices (no response given within 3 s of food pairs presentation). All these variables were normally distributed except for the missed choice percentage and thus, to obtain a normal distribution, we applied a fractional rank transformation (Mahachie John et al., 2013) ranging from 0 (lowest fractional rank) to 1 (largest fractional rank). In order to assess the effect of tRNS condition on the HC food choice, three (one for each dependent variable) repeated measures analysis of variance (ANOVAs) were performed. ANOVAs with percentage of HC food choices and missed choices rank as dependent variables had the following factors: tRNS condition (lSTS, SH, rSTS) and gender (female = F, male = M). The ANOVA on RTs had in addition the factor food choice (HC, LC). One participant showing outlier values (criterion: ± 3 SD) was excluded.

Finally, given the variability of stimulation time across our sample, we controlled for possible effects deriving from different stimulation time. Thus, a series of *T*-test between a first group of trials (i.e., trials presented between ~150 s and ~350 s of total stimulation) and a second group of trials (i.e., trials presented between ~350 s and ~650 s of total stimulation) were performed on HC food choices, RTs and percentage of missed choices for all stimulation conditions (lSTS, SH, rSTS). For all comparisons, no significant effect was found (for all cases *p* > 0.05).

## Results

**Figure 3:**
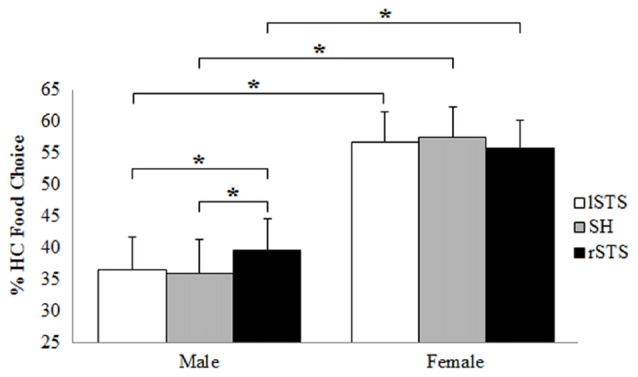
Interaction between tRNS condition and participants gender on % of high-calorie (HC) food choice (lSTS, left posterior STS stimulation; SH, Sham control condition; rSTS, right posterior STS stimulation). Data are presented as mean values + standard errors. *p < 0.05.

Considering the percentage of HC food choice as dependent variable, ANOVA with tRNS condition (lSTS, SH, rSTS) and participants gender (F, M) as factors showed a significant effect of gender (*F*_(1,32)_ = 7.67, *p* = 0.009, $\eta_{\text{p}}^{\text{2}}$ = 0.193) and a significant interaction tRNS × Gender (*F*_(2,64)_ = 4.23, *p* = 0.018, $\eta_{\text{p}}^{\text{2}}$ = 0.117). Duncan *post hoc* analysis showed (Figure 3) that females chose more HC foods compared to males for SH condition (*p* = 0.009), lSTS (*p* = 0.011) and rSTS (*p* = 0.029) stimulation. In males, the percentage of HC choices was higher during rSTS stimulation compared to SH condition (*p* = 0.013) and lSTS stimulation (*p* = 0.028).

Considering the median of RTs to food choice as dependent variable, ANOVA with tRNS condition (lSTS, SH, rSTS), food choice (HC, LC) and gender (F, M) as factors showed no significant effects.

**Figure 4:**
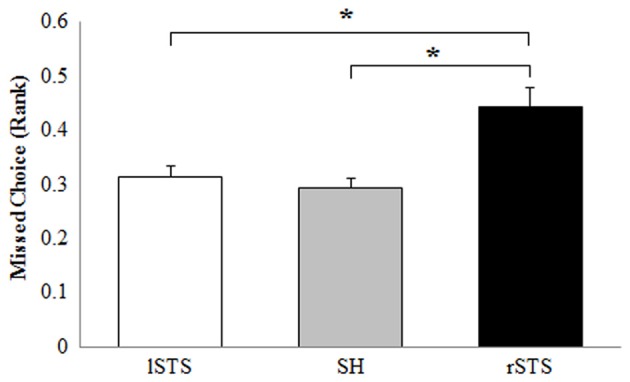
Effect of tRNS condition on rank transformed % of missed choices (lSTS, left posterior STS stimulation; SH, Sham control condition; rSTS, right posterior STS stimulation). Data are presented as mean values + standard errors. *p < 0.05.

Considering the rank transformed percentage of missed choice as dependent variable, ANOVA with tRNS condition (lSTS, SH, rSTS) and participants gender (F, M) as factors showed a significant effect of tRNS (*F*_(2,64)_ = 11.44, *p* < 0.001, $\eta_{\text{p}}^{\text{2}}$ = 0.263) and no other significant effect. Duncan *post hoc* analysis showed (Figure 4) more missed choices for rSTS stimulation compared to SH condition (*p* < 0.001) and lSTS stimulation (*p* < 0.001).

## Discussion

In the present study, we tested the hypothesis that HF tRNS on the right posterior STS causally influences food choices between equally valenced high and low caloric foods in sated individuals. Given the gender differences in food preference and brain responses to food (e.g., Del Parigi et al., 2002; Wardle et al., 2004; Smeets et al., 2006; Killgore and Yurgelun-Todd, 2010; Haase et al., 2011; Arganini et al., 2012), we aimed to assess gender differences in the effects of left and right STS stimulation and sham condition on food choice. Male participants showed a general preference for LC food compared to women. Moreover, we found an increase of HC choice in male participants, but not in women, during the tRNS of posterior right STS compared to sham condition and left tRNS. Finally, although no effect was found with regard to RTs, we found, in both males and females, an increase of missed choices when posterior right STS was stimulated compared to sham condition and left stimulation.

Regular eating of HC foods in the absence of hunger might lead to a positive energy balance and overweight on the long term. As showed by our rating data, low energy foods are perceived to be healthier and less heavy compared with high energy foods (Deng and Kahn, 2009; Charbonnier et al., 2016; Prada et al., 2017). Thus, choosing LC foods when sated might be beneficial to maintain a stable weight. Despite that, in our study only male participants showed this tendency. In fact, our female sample showed the opposite tendency, namely a greater rate of HC food choice. This result could be explained by the “Reflective-Impulsive Model” (Strack and Deutsch, 2014), according to which reflective system generates behavioral decisions based on knowledge, facts and values, whereas the impulsive system elicits behavior through associative links and motivational orientations. It has been stated that women, more than men, fail to act upon the cognitive conflict between eating enjoyment and weight control/healthy issue (e.g., Gough and Conner, 2006; Ree et al., 2008). Thus, although women are generally more healthy-oriented (Wardle et al., 2000; Cooke and Wardle, 2005; Shiferaw et al., 2012), when they are stressed and a more tempting alternative is concurrently available, females produce a more impulsive reward-oriented food choice compared to men (Hofmann et al., 2007; Stroebe et al., 2008; Myrseth et al., 2009; van der Laan et al., 2014), choosing mainly sweet snacks and increasing their food intake (Wansink et al., 2003; Kandiah et al., 2006). Actually, females resulted as more involved in self-body issue and showed higher emotional eating scores compared to males, in line with the current bibliography (e.g., van Strien et al., 1986; Pingitore et al., 1997; Marano et al., 2007; Dakanalis et al., 2013).

This conflict has been analyzed by a neuroimaging study of Killgore and Yurgelun-Todd (2010). They found a significantly greater activation to HC foods within a distributed system of lateral prefrontal and midline cortical regions involved in cognitive analysis (Pochon et al., 2001), behavioral control (Leung and Cai, 2007) and self-referential cognition (Moran et al., 2009; D’Argembeau et al., 2010) in women. On contrary, men showed larger amygdala responses, the primal limbic structure involved in detecting biologically relevant stimuli and in determining the appetitive value or attractiveness of food (Piech et al., 2009). These findings are consistent with the gender differences in responses to food and feeding behaviors, namely a lower level of cognitive conflict and guilt in men compared to women (Rolls et al., 1991; Narchi et al., 2008). Therefore, the bidirectional connections of STS (Seltzer and Pandya, 1989a,b, 1994; Barnes and Pandya, 1992; Hein and Knight, 2008) with a variety of prefrontal and limbic structures (Cardinal et al., 2002; Salzman and Fusi, 2010; Delli Pizzi et al., 2015, 2016) could explain the different effect of right STS stimulation between males and females. Particularly, the increase of excitability of right STS produced by HF tRNS (Terney et al., 2008) could have modulated the brain activity in order to promote the HC choice in men but not in women, may be due to a preponderant activity of prefrontal areas in women and of STS in men. An alternative explanation could rely on different menstrual cycle phases of our female participants. For examples, Gorczyca et al. (2016) reported an increase of protein intake and food cravings during the luteal phase. Despite that, independently from participants sex, the tRNS of right posterior STS induced a highly degree of indecision revealed by the missed choice increase, confirming the involvement of this area in this kind of decision.

While different studies using tDCS showed that the dorsolateral prefrontal cortex modulated food evaluation, desire and consumptions (Fregni et al., 2008; Camus et al., 2009; Lapenta et al., 2014) no one study had focused on the effect of STS stimulation in this field. Generally, the STS is thought to be a multifunctional region ranging from facial recognition to social cognition and theory of mind (Hein and Knight, 2008). Specifically, the right STS have been linked to eating behavior and eating disorder in several studies. Prior to the study of Charbonnier et al. (2015) in which this area exhibited greater activation when participants chose HC food compared to LC ones, Coletta et al. (2009) found a greater right posterior STS activity during viewing highly palatable (energy rich) compared to moderately palatable foods in fasted state volunteers. Furthermore, Braun and Chouinard (1992) showed that anorexia is frequently associated with right posterior Hypometabolism (e.g., occipito-temporo-parietal junction) and right anterior hypermetabolism, both associated with right-sided abnormal electroencephalogram spiking. Finally, the left vagal stimulation seems to induce different brain response such as a significant increase of blood flow in right STS (Conway et al., 2012; Manta et al., 2013). The vagus nerve plays a key role in the homeostatic control of food intake, regulating the bidirectional communication between the periphery (digestive system) and the central nervous system (Williams and Elmquist, 2012).

### Limitations and Future Directions

Our results are limited to a young adult sample; thus, given the age effect on food perception (e.g., Kremer et al., 2007; Padulo et al., 2017) and in brain function and structure (Volkow et al., 1998; Sowell et al., 2003), future research could investigate these mechanism in different age groups (as adolescent or elderly). A limitation of our study is that we did not measure in which phase of the menstrual cycle female participants where. Therefore, we cannot rule out that menstrual cycle may have influenced our results (e.g., Gorczyca et al., 2016). Regarding the stimulation, it is important to bear in mind that transcranial electrical stimulation has low spatial resolution and that individual anatomical differences could exert differential effects in the current flow thus leading to possibly different results (Neuling et al., 2012). In addition, brain stimulation studies should be interpreted with caution, since whereas shared involvement of active/stimulated areas can imply actual common roles, the directions of the observed effects do not allow to reliable speculations. Finally, we had no clear reference about the kind and the timing of the effects exerted by HF-tRNS stimulation on STS during our task, as well as we can not state that the effects here observed on food choice are pure and did not influence (or were influenced by) other cognitive/emotional processes in our participants (for a review see Hein and Knight, 2008). Further studies keeping in control other psychological dimensions could say a bit more on these possible interactions. On the other side, it should be remarked that stimulation techniques allow to test in a more precise way the relation between brain activity and behavior, in comparison to neuroimaging techniques. Indeed, while the latter do not set apart behavior-effective from side activity, effects due to brain stimulation insist on processes which cause the observed behavior. In the current study we assessed short-term effects of tRNS. Future study could investigate long lasting/off-line effects of tES on food choice. Our finding, that right STS on-line stimulation increased preference for HC foods, suggest that tRNS can influence food choice. It may be that stimulation of other areas, e.g., those involved in control, could decrease preference for unhealthy foods. Further research could investigate possible effects of transcranial stimulation in healthy and clinical (e.g., obese and anorexia nervosa) populations on food choice to assess the potential of these techniques in eating disorders therapy.

## Conclusion

In conclusion, we confirm the involvement of right posterior STS in feeding behavior and assign to its activity a possible causal role in modulating this function. As a novel finding, we report a difference between men and women indicating a prominent role of STS in HC/LC food choice confirming the existence of different neuro-cognitive reward pattern based on sex.

## Author Contributions

VM, CP and AB conceived and designed the experiment. VM collected and analyzed the data. VM, CP, LNL and AB interpreted the data. VM wrote the manuscript and CP, LNL and AB provided critical revisions and contributed to the final version of the manuscript by interpreting results, reviewing and critically revising text. All authors approved the final version for submission and agreed to be accountable to for all aspects of the work.

## Conflict of Interest Statement

The authors declare that the research was conducted in the absence of any commercial or financial relationships that could be construed as a potential conflict of interest. The reviewer BW and handling Editor declared their shared affiliation.

## Footnotes

^1^http://nutritionalneuroscience.eu
